# Impact of Arachidonic and Docosahexaenoic Acid Supplementation on Neural and Immune Development in the Young Pig

**DOI:** 10.3389/fnut.2020.592364

**Published:** 2020-10-29

**Authors:** Kaylee E. Hahn, Irina Dahms, Christopher M. Butt, Norman Salem, Vivian Grimshaw, Eileen Bailey, Stephen A. Fleming, Brooke N. Smith, Ryan N. Dilger

**Affiliations:** ^1^Piglet Nutrition & Cognition Laboratory, Department of Animal Sciences, University of Illinois, Urbana, IL, United States; ^2^Division of Nutrition Sciences, University of Illinois, Urbana, IL, United States; ^3^DSM Nutritional Products, Kaiseraugst, Switzerland; ^4^Bolder BioPATH, Inc., Boulder, CO, United States; ^5^DSM Nutritional Products, Columbia, MD, United States; ^6^Neuroscience Program, University of Illinois, Urbana, IL, United States

**Keywords:** arachidonic acid (ARA), docosahexaenoic acid (DHA), pediatric nutrition, comparative nutrition, pig, polyunsaturated fatty acid (PUFA), neural, immune

## Abstract

**Background:** Human milk contains both arachidonic acid (ARA) and docosahexaenoic acid (DHA). Supplementation of infant formula with ARA and DHA results in fatty acid (FA) profiles, neurodevelopmental outcomes, and immune responses in formula-fed infants that are more like those observed in breastfed infants. Consequently, ARA and DHA have been historically added together to infant formula. This study investigated the impact of ARA or DHA supplementation alone or in combination on tissue FA incorporation, immune responses, and neurodevelopment in the young pig.

**Methods:** Male pigs (*N* = 48 total) received one of four dietary treatments from postnatal day (PND) 2–30. Treatments targeted the following ARA/DHA levels (% of total FA): CON (0.00/0.00), ARA (0.80/0.00), DHA (0.00/0.80), and ARA+DHA (0.80/0.80). Plasma, red blood cells (RBC), and prefrontal cortex (PFC) were collected for FA analysis. Blood was collected for T cell immunophenotyping and to quantify a panel of immune outcomes. Myelin thickness in the corpus callosum was measured by transmission electron microscopy and pig movement was measured by actigraphy.

**Results:** There were no differences in formula intake or growth between dietary groups. DHA supplementation increased brain DHA, but decreased ARA, compared with all other groups. ARA supplementation increased brain ARA compared with all other groups but did not affect brain DHA. Combined supplementation increased brain DHA levels but did not affect brain ARA levels compared with the control. Pigs fed ARA or ARA+DHA exhibited more activity than those fed CON or DHA. Diet-dependent differences in activity suggested pigs fed ARA had the lowest percent time asleep, while those fed DHA had the highest. No differences were observed for immune or myelination outcomes.

**Conclusion:** Supplementation with ARA and DHA did not differentially affect immune responses, but ARA levels in RBC and PFC were reduced when DHA was provided without ARA. Supplementation of either ARA or DHA alone induced differences in time spent asleep, and ARA inclusion increased general activity. Therefore, the current data support the combined supplementation with both ARA and DHA in infant formula and raise questions regarding the safety and nutritional suitability of ARA or DHA supplementation individually.

## Introduction

Infants exhibit rapid growth and development during early life and require adequate intake of critical nutrients for optimal health outcomes. Arachidonic acid (ARA, 20:4n-6) and docosahexaenoic acid (DHA, 22:6n-3) are long-chain polyunsaturated fatty acids (LCPUFA) that are present in breast milk and have important structural and physiological roles in early development. Global average levels of ARA and DHA in breast milk (% of total FA by weight) are estimated to be 0.47 ± 0.13 and 0.32 ± 0.22%, respectively (1). While infants can endogenously synthesize ARA and DHA from their precursors, linoleic (LA; 18:2n-6) and alpha-linolenic (ALA; 18:3n-3) fatty acids, respectively, those receiving infant formula (IF) supplemented with both ARA and DHA exhibit tissue FA accretion, as well as cognitive, visual, and immune outcomes that are more similar to those reported for breastfed infants (2–9). Additionally, polymorphisms in the fatty acid desaturase (FADS) genes influence ARA and DHA concentrations, and infants with specific genotypes may require higher levels of these fatty acids (FA) to maintain an adequate status. Depending on the infant's genotype, IF supplementation might not be sufficient for all infants to narrow the gap of ARA and DHA concentrations between breastfed and formula-fed infants (10).

As such, combined ARA and DHA supplementation in IF has been generally implemented to mimic breast milk, as well as more closely match outcomes in breastfed infants and manage FADS genotypic differences among infants. While the need for DHA supplementation in IF has been well-established due to positive impacts on retinal and cognitive outcomes, the need for ARA supplementation has been less well-documented. Nevertheless, experts and regulatory bodies generally agree about the need for combined ARA and DHA supplementation, with a need for equal or greater amounts of ARA when DHA is provided (4, 11–13). Although optimal ARA:DHA ratios are not fully elucidated, global breast milk levels reflect the importance of balancing both LCPUFA in the infant's diet, due to possible impacts on the immune response, the risk for atopic disease, cognitive and behavioral outcomes, and competition for tissue incorporation (4, 14–18). Newly adopted regulatory standards on IF for the European Union require that IF contain 20–50 mg DHA/100 kcal of milk, equivalent to about 0.5–1% of total fatty acids and higher than worldwide breast milk averages, without a requirement for ARA. This has incited considerable concern due to the lack of evidence on the suitability and safety of this novel IF composition in healthy infants. Indeed, a recently published expert opinion raised several concerns regarding supplementing formulas for infants with DHA but without ARA (12). These concerns included the possibility of undesirable outcomes such as decreased concentrations of ARA in the brain, as well as potential negative impacts on neurodevelopment, growth, and immunity.

Much of the focus surrounding ARA and DHA in early development has centered on functions within the neural and immune systems. Both FA rapidly accumulate in the central nervous system during the third trimester and the first years of life, thereby representing the two most abundant polyunsaturated fatty acids (PUFA) in the retina and brain (19–21). The connection between these PUFA and neural development involves myelination trajectories, where structure and function meet. DHA plays a particularly important role in the central nervous system, where it is involved in neurotransmission, neurogenesis, and protection from oxidative stress (22–24). In clinical trials, the addition of ARA and DHA to IF has been shown to improve measures of cognitive development and visual acuity (25–28). Improvements in cognitive development and visual acuity may involve myelination, which can be accurately quantified using microscopy techniques. Infants provided IF with a combined supplementation of ARA and DHA during the first 17 weeks of life yielded comparable visual acuity and verbal intelligence quotient (IQ) scores to that of breastfed infants at 4 years of age; however, DHA alone only achieved similar visual acuity (6). Cognitive benefits from combined ARA and DHA supplementation in early life have also been observed through early and middle childhood (29, 30). The balance between these LCPUFA is likely important for cognitive outcomes, as the cognitive benefits observed in infants receiving supplemented formula were shown to be reduced when DHA to ARA was provided at a 1.5:1 ratio compared with that of infants receiving a ratio of 1:1 or 1:2 (14). Intake of ARA and DHA has also been shown to influence additional neural related outcomes including sleep, myelination, and motor activity (31–38).

The need for dietary inclusion of both ARA and DHA is also emphasized in regard to immune system response and development. Inhuman peripheral blood mononuclear cells (PBMC), ARA usually constitutes ~19–23% of total FA, whereas highly unsaturated n-3 FA (primarily DHA) comprise only ~2–3% (39). Diet-induced alterations in these concentrations are believed to influence immune cell membrane structure and functions. Supplemental ARA and DHA have been shown to modulate T-cell functions, cytokine profiles, eicosanoid synthesis, and possibly B cell activation (40–43). ARA serves as a precursor for predominantly pro-inflammatory eicosanoids, while DHA serves as a precursor for anti-inflammatory resolvins, docosatrienes, and protectins (40, 41). Compared with an unsupplemented IF, those supplemented with ARA and DHA have been shown to elicit immune responses more comparable to that of a breastfed infant (44). While reducing excessive inflammation is often beneficial, the inflammatory response is ultimately a protective mechanism, and over suppression may be disadvantageous during early life when an infant is still developing the ability to produce an appropriate and efficient immune response (4, 15).

Anatomic, physiologic, immunologic, and metabolic similarities make the young pig an ideal preclinical model for studying aspects of infant development and growth (45–51). The young pig has strikingly similar nutrient requirements to humans during infancy and growth (52) and humans share more immune-related genes and proteins with pigs than mice (53). Rapid growth and similarities in gastrointestinal physiology and metabolism also make the pig a particularly attractive model for nutritional intervention studies (47). Additionally, the morphology and peak brain growth of the pig more closely resembles that of humans, in which the total brain volume growth of a one-month-old human is equivalent to that of a one-week-old pig (48, 54). While the conversion of essential fatty acids to ARA and DHA are less clearly defined in the pig compared with rodents, the pig model provides more comparable essential FA metabolism to that of humans (55, 56). Thus, the young pig model has been instrumental in advancing preclinical research for infant nutrition and development.

The impact of independent ARA and DHA supplementation is poorly understood. There is a dearth of literature evaluating the impact of IF with DHA alone on immune response, and few studies have assessed the impact of ARA alone on any outcome. Against this and the above background, the primary objective of this study was to use the young pig model to evaluate the impact of individual or combined supplementation of ARA and DHA on growth, tissue FA accretion, the development of appropriate immune responses, and motor activity. We hypothesized that providing both ARA and DHA would positively impact immune functions, and result in a more robust immune response compared with DHA alone. We also hypothesized that the provision of DHA at this level, in the absence of dietary ARA, would reduce endogenous concentrations of ARA in pertinent tissues.

## Materials and Methods

### Animal Care and Housing

Forty-eight naturally-farrowed intact male pigs were obtained from a commercial swine farm (PIC 1050 genetics; Carthage Veterinary Service, Ltd., Carthage, IL) and transported to the Piglet Nutrition and Cognition Laboratory (PNCL) on postnatal day (PND) 2. All pigs received a single prophylactic antibiotic injection on PND 1 (Excede; Zoetis, Parsippany, NJ), and were administered 5 mL of Clostridium perfringens types C and D antitoxin subcutaneously and orally upon entering PNCL on PND 2 as a prophylactic measure to avoid enterotoxemia that sometimes occurs in artificially-reared pigs. The study was completed in two cohorts, with 12 pigs per treatment total (six pigs per treatment per cohort). Pigs were selected across 12 litters to control for genetic variation, with pigs from each litter distributed across dietary treatment groups. If pigs experienced diarrhea lasting three or more days, they were administered an oral electrolyte solution (Bounce Back; Manna Pro Products, Chesterfield, MO) to maintain fluid and electrolyte balance. Pigs were housed individually in cages constructed of clear polycarbonate and stainless steel with flooring consisting of vinyl-coated expanded metal designed for young pigs. These caging units allow for urine and feces to pass through the flooring and flow into septic lines. Cages were maintained in a climate-controlled room kept between 28 and 30°C. The rearing environment was maintained on a 12 h light and dark cycle from ~0800 to 2000 h. Cage dividers were clear and perforated, allowing pigs to see, hear, and smell each other, but not directly touch. All pigs were provided with environmental enrichment (e.g., toys) and a cotton towel for comfort. Pigs were artificially reared until PND 30 then humanely euthanized via CO_2_ asphyxiation. All animal care and experimental procedures were in accordance with the National Research Council Guide for the Care and Use of Laboratory Animals and approved by the University of Illinois at Urbana-Champaign Institutional Animal Care and Use Committee.

### Dietary Treatments and Feeding

Custom milk replacer (MR) products (TestDiet; St. Louis, MO) were formulated to be nutritionally-adequate for young pigs (57). The MR formulas were based on soy protein isolate to ensure no inherent contribution of either ARA or DHA; internal FA analyses of commercial pig whey-based MR powders revealed moderate levels of ARA (~0.1–0.3% total FA; data not shown). Powdered fat products included in experimental MR were selected after internal analyses verified they were devoid of ARA and DHA (data not shown). Pigs were randomly allotted (*n* = 12 per treatment) to one of four isocaloric experimental milk replacers (Table 1) by initial body weight and litter using the Experimental Animal Allotment Program (58). Supplemental ARA and DHA were provided in the form of single-cell oils (ARASCO^TM^ and DHASCO^TM^, respectively; DSM Nutritional Products, Colombia, MD) at concentrations reflecting feasible upper levels allowed via supplementation, albeit slightly higher than what may typically be expected in breast milk (1, 59). Both MR and fresh water were provided *ad libitum* throughout the study. Each day, MR powder from each treatment was reconstituted with 200 g of MR powder per 800 g water and dispersed via an automated liquid feeding system for a 20 h cycle, with the remaining 4 h period used to clean components of the feeding system and introduce fresh MR.

**Table 1:** Nutrient concentrations of dietary treatments^a^.

	**Dietary treatment**			
**Item**	**CON**	**ARA**	**DHA**	**ARA+DHA**
**Ingredients, g/kg**				
Lactose	40.40	40.40	40.40	40.40
Soy protein isolate^b^	25.32	25.32	25.32	25.32
Died coconut oil^c^	15.30	15.06	15.06	14.82
Dried MCT oil^d^	7.65	7.53	7.53	7.41
Dicalcium phosphate	2.00	2.00	2.00	2.00
Calcium carbonate	1.98	1.98	1.98	1.98
Potassium citrate tribasic monohydrate	1.88	1.88	1.88	1.88
Vitamin and mineral premix^e^	1.17	1.17	1.17	1.17
Salt	1.13	1.13	1.13	1.13
Potassium sorbate	1.00	1.00	1.00	1.00
Lecithin	0.90	0.90	0.90	0.90
L-Lysine	0.51	0.51	0.51	0.51
ARASCO^f^	0.00	0.36	0.00	0.36
DHASCO^f^	0.00	0.00	0.36	0.36
Choline chloride	0.24	0.24	0.24	0.24
L-Cystine	0.20	0.20	0.20	0.20
DL-Methionine	0.15	0.15	0.15	0.15
Powdered cellulose	0.09	0.09	0.09	0.09
Palatant^g^	0.08	0.08	0.08	0.08
**Nutritional profile^h^**				
Energy, kcal/g	4.36	4.37	4.37	4.37
Carbohydrates, %	43.7	43.6	43.6	43.5
Protein, %	24.5	24.5	24.5	24.4
Fat, %	18.2	18.3	18.3	18.4
Linoleic acid, %	0.68	0.68	0.68	0.67
Linolenic acid, %	0.02	0.02	0.02	0.02
SFA, %	15.89	15.64	15.64	15.39
MUFA, %	0.73	0.72	0.72	0.70
PUFA, %	0.14	0.28	0.28	0.42
**Analyzed Composition, % of Total FA^i^**				
ARA	0.00 (0.00)	1.15 (0.80)	0.00 (0.00)	0.79 (0.80)
DHA	0.00 (0.00)	0.00 (0.00)	0.89 (0.80)	0.78 (0.80)

a *Diets were manufactured as custom blends formulated by TestDiet (St. Louis, MO). All pigs received allotted treatment from PND 2 to PND 30. ARA, arachidonic acid; DHA, docosahexaenoic acid; CON, control; MCT, medium chain triglycerides; FA, fatty acid; MUFA, monounsaturated fatty acids; SFA, saturated fatty acids; PUFA, polyunsaturated fatty acids; PND, postnatal day*.

b *Ardex F, Archer Daniels Midland, Decatur, IL*.

c *Centennial 72 Coconut IP2, Sensory Effects, Defiance, OH*.

d *Vital Blend MCT NG, Sensory Effects, Defiance, OH*.

e *Custom vitamin and mineral premix. Provided per gram of complete diet: Ca (12.8 mg), P (7.8 mg), K (10 mg), Mg (1 mg), Na (8.7 mg), Cl (8.5 mg), F (8.1 mcg), Fe (161 mcg), Zn (100 mcg), Mn (46 mcg), Cu (19.2 mcg), Co (0.6 mcg), I (1.18 mcg), Mo (1.02 mcg), Se (0.3 mcg), Vitamin B12 (0.11 mcg), Vitamin K (5 mcg), thiamin (2.7 mcg), riboflavin (13.5 mcg), niacin (60 mcg), pantothenic acid (30 mcg), folic acid (1 mcg), pyridoxine (3 mcg), biotin (0.3 mcg), choline chloride (2.06 mg), ascorbic acid (49.2 mcg), Vitamin A (2.8 IU), Vitamin D3 (6.7 IU), Vitamin E (0.33 IU)*.

f *ARASCO and DHASCO, DSM Nutritional Products, Heerlen, Netherlands*.

g *Luctarom Milky Vanilla, Lucta, Barcelona, Spain*.

h *Based on calculated values from latest ingredient analysis information provided by TestDiet. Nutrients expressed as percent of diet on an as-fed basis*.

i *Target fatty acid concentrations are shown in brackets*.

### Immune Stimulation

Following a 1-week adaptation period to the facility, all pigs received a two-dose series of keyhole limpet hemocyanin [KLH; hemocyanin from *Megathura crenulate* (keyhole limpet), CAS: 9013-72-3; Sigma Aldrich, Saint Louis, MO] via intraperitoneal injection (Becton Dickinson & Company Precision Glide Needle 22 gauge × 2.54 cm, Cat No: 305155). The first dose (1 mL at 10 mg of KLH/mL) was administered on PND 9 and the second (1 mL at 2 mg of KLH/mL) on PND 19.

### Growth Performance and Wellness

Individual pig and MR hopper weights were recorded daily to calculate average daily body weight gain (ADG) and average daily milk intake (ADMI; net disappearance of MR), respectively. Health checks occurred twice daily, and stool consistency was visually assessed and scored daily using the following scale: 1 = solid; 2 = semisolid; 3 = loose; 4 = watery. Rectal temperatures were measured on PND 9, 12, 16, 19, 24, 26, and 30 using a digital thermometer (GLA Agricultural Electronics, San Luis Obispo, CA). On days when KLH injections or blood collections occurred, rectal temperatures were recorded before those procedures.

### Pig Activity

Twenty-one pigs from the first cohort (*n* = 5 or 6 per treatment) were fitted with adjustable collars bearing actigraphy monitors containing an accelerometer (Actiwatch 2; Philips Respironics, Bend, OR) to quantify average movement and approximate sleep and wake activity using previously validated and described protocols (60).

### Immune Analyses

Whole blood was collected on PND 16, 26, and 30 into 4-mL evacuated K_2_EDTA blood tubes (Becton Dickinson & Company, Cat No: 367835) using 21 gauge × 3.18 cm collection needles (Becton Dickinson & Company, Cat No: 368607). Samples were placed on ice until centrifuged to separate plasma and RBC (4°C, 1,250 × g, 15 min; Allegra 6R centrifuge, Beckman Coulter Life Sciences, Indianapolis, IN). Both were aliquoted and stored at −80°C. Plasma from PND 16 and 26 was analyzed for anti-KLH IgG antibodies [Porcine Keyhole Limpet Hemocyanin Antibody IgG (KLH-IgG) ELISA kit, Cat. No: MBS9364989; MyBioSource, San Diego, CA]. A preliminary anti-KLH IgG assessment was conducted using samples of plasma (*n* = 17) and serum (*n* = 16) collected from non-KLH-exposed pigs originating from the same swine farm and raised under identical rearing conditions. This revealed an average of 1.32 ng/mL of background anti-KLH IgG in non-KLH-exposed pigs. Thus, KLH antibody values obtained for this trial were corrected based on this value prior to statistical analyses to account for baseline KLH antibody presence.

Plasma from PND 16 and 26 was analyzed for circulating cytokines IL-1β, IL-10, IFN-α, IFN-γ, TNF-α, IL-4, and IL-8 (Invitrogen Swine Cytokine Magnetic 7-Plex Panel, Cat No: LSC0001M, Novex® by Life Technologies, Frederick, MD). Plasma from PND 30 was used to quantify circulating interleukin (IL)-17A (Porcine IL-17A ELISA Kit, catalog number ESIL17A; Thermo Fisher Scientific, Frederick, MD), thromboxane-B2 (Porcine TXB2 ELISA Kit, catalog number MBS036106; MyBioSource, San Diego, CA), and prostaglandin E2 (PGE2 ELISA Kit, catalog number MBS2884477; MyBioSource, San Diego, CA).

After plasma was removed from whole blood on PND 16 and 26, the remaining sample was used to isolate PBMC for T cell immunophenotyping via flow cytometry. The sample was placed over a density gradient (SepMate-15 [IVD] and Lymphoprep, StemCell Technologies, Cambridge, MA, Cat. No: 85415 and 07851) and centrifuged (20°C, 1,200 × g, 20 min). Isolated PBMC were separated and washed with phosphate buffer saline containing 2% fetal bovine serum (Thermo Fisher Scientific, Cat. No: 10082147). PBMC were rinsed once with Ammonium Chloride Lysis Solution (StemCell Technologies, Cat. No: 07850). Cells were counted (Moxi Z Mini Automated Cell Counter; ORFLO Technologies, Ketchum, ID) and diluted to 1.0 × 10^6^ cells/mL. Samples were labeled with external fluorescent antibodies against cell surface markers CD3 (FITC Mouse Anti-Pig CD3ε, Cat 559582, Lot 8248740, Becton Dickinson & Company Pharmingen, San Jose, CA), CD4 (Alexa Fluor® 647 Mouse Anti-Pig CD4a, Cat 561472, Lot 8334871, Becton Dickinson & Company Pharmingen, San Jose, CA), and CD8 (PE Mouse Anti-Pig CD8b, Cat 561484, Lot 9073659, Becton Dickinson & Company Pharmingen, San Jose, CA). CD8 was diluted using BD Brilliant Stain Buffer (Becton Dickinson & Company Horizon, Cat No. 563794). Labeled cells were fixed using 4% paraformaldehyde [Pierce™ 16% Formaldehyde (w/v), Methanol-free, Thermo Fisher Scientific, Cat No: 28908] and analyzed at the University of Illinois Roy J. Carver Biotechnology Center Flow Cytometry Facility using a BD LSR II Flow Cytometry Analyzer (Becton Dickinson & Company Biosciences, San Jose, CA). Outputs were analyzed using FCS Express 5 Plus (*De Novo* Software, Glendale, CA). The number of CD3^+^ events constituted the total number of T cells, while CD3^+^CD4^+^CD8^−^, CD3^+^CD4^−^CD8^+^, and CD3^+^CD4^+^CD8^+^, were considered helper, cytotoxic, and memory T cells, respectively.

### Hematological Outcomes

Whole blood, plasma, and serum samples were collected on PND 30 and submitted to the University of Illinois College of Veterinary Medicine Clinical Pathology Laboratory for analysis of blood coagulation factors (PT/PTT/Fib), complete blood cell counts with differentials (CBC), and general health serum chemistry. Whole blood for CBC and PT/PTT/Fib were collected into 4-mL evacuated K_2_EDTA and 1.8-mL sodium citrate 3.2% blood tubes, respectively, and stored on ice prior to submission (Becton Dickinson & Company, Cat No: 367835 and 363080). Serum samples were collected into 4-mL serum tubes (Becton Dickinson & Company, Cat No: 367812) and left at room temperature to clot for a least 30 min. All samples were processed and stored at −80°C within 6 h of collection.

### Tissue Collection

Organ weights and tissue samples were collected on PND 30, including liver, whole brain, spleen, thymus, duodenum, jejunum, and ileum. Tissue aliquots were snap-frozen in liquid nitrogen and stored at −80°C. Total small intestine length and individual section weights were also recorded. The first 10% of the total length was considered duodenum, the last 15% ileum, and the remainder jejunum.

### Fatty Acid Analyses

Tissue FA (plasma, PFC, and RBC) were quantified by gas chromatography. Briefly, plasma was dried under nitrogen, PFC was lyophilized, homogenized and weighed, and RBC were vortexed and aliquoted directly. Internal standard (trinonadecanoic acid or pentadecanoic acid in toluene) was added to each sample, and 1.5 N methanolic hydrochloric acid was used for direct transesterification. Samples were heated for 2 h at 100°C. Following methylation, saturated sodium chloride was added, and lipids were extracted into toluene for direct injection. Calibration curves were generated using GLC-502B (Nu-Chek Prep, Elysian, MN) for FA reference standards. Samples were analyzed on an Agilent 6890 gas chromatographer (split injection) equipped with a name ionization detector. A 30 m × 0.32 mm × 0.2 μm SP-2380 fused silica capillary column (Supelco, Bellefonte, PA) was used with hydrogen as the carrier gas. Oven temperature was programmed from 140 to 190°C at 5°C/min, held for 1 min at 190°C, increased to 260°C at a rate of 17°C/min, then held for 3 min for a total run time of 18.12 min. The name ionization detector was set at 285°C. FA concentrations are expressed as weight percent of total FA.

### Brain for TEM

Left brain hemispheres from the second cohort of pigs (*n* = 3 per treatment group) were prepared for the measurement of axonal myelination in the genu of the corpus callosum via TEM. Immediately following euthanasia, whole hemispheres were submerged in 4% PFA for 1 week, then transferred to 0.15M cacodylate buffer (pH 7.4) containing 2.5% glutaraldehyde, 2% PFA and 2 mM CaCl_2_ at 5–8°C. The corpus callosum was chosen because of its proximity to the frontal lobe, its postnatal myelination pattern, and the ability to achieve adequate fixation without impacting primary outcomes. After initial fixation, the genu was dissected away from the rest of the hemisphere, rinsed with 3, 15-min washes in 0.1 M sodium cacodylate and post-fixed for 2 h in 2% osmium tetroxide in 0.1 M sodium cacodylate. The tissue was then rinsed again three times for 15 min in 0.1 M sodium cacodylate and placed in fresh 0.1 M sodium cacodylate overnight at 4°C. The following day, the tissue was washed three times for 15 min in deionized water at room temperature. It was then dehydrated at room temperature through the following steps: 30 min each in 25, 50, 75, 95, and 100% ethanol (EtOH) in water, 1 h in fresh 100% EtOH, 5 min in 50% propylene oxide/50% EtOH, 5 min in 100% propylene oxide. The tissue was then embedded in epoxy resin, incubated for 48 h at 60°C, oriented in a manner that resulted in the majority of axons being sliced orthogonally in the sagittal plane, and sliced with a Leica Ultracut UCT ultramicrotome set at 50 nm. The slices were mounted in imaging grids and stained with 4% uranyl acetate in methanol and then with Reynold's lead citrate (61). Images were captured with a FEI Tecnai T12 Spirit electron microscope at 5,000× magnification. Images were analyzed with Icy imaging software (http://icy.bioimageanalysis.org/), and analysis consisted of tracing the circumferences of both the axonal membrane and the outer bound of the myelin sheath for 300–400 individual axons per animal. Axon diameter, myelin thickness, and the G-ratio were then calculated from the circumference values.

### Intestinal Structure

Intestinal macrostructure (i.e., villus height, crypt depth, and mucosal thickness) was evaluated by a board-certified histopathologist (Veterinary Diagnostic Pathology, LLC, Fort Valley, VA). Evaluations were made using 5-micron Hematoxylin and Eosin stained sections prepared from formalized samples of duodenum, jejunum, and ileum (formalin solution, neutral buffered, 10%, Sigma-Aldrich, HT501128). Microscopic examinations included semi-quantitative severity scoring of both histopathology lesions and microscopic histologic features using the following scoring system: 0 = absent; 1 = minimal; 2 = mild; 3 = moderate; 4 = marked; and 5 = severe. Half values were given when uncertainty existed between scoring groups in assigning a value. Villus/crypt ratios and total mucosal thickness were calculated using five well-oriented villi and crypt measurements per intestinal tissue sample.

### Statistical Analysis

For single time-point outcomes, a 1-way ANOVA was conducted using the MIXED procedure of SAS 9.4 (SAS Institute, Inc., Cary, NC) with the fixed effect of diet and the random effect of cohort. For repeated immune measures, a 2-way ANOVA was conducted using the MIXED procedure of SAS 9.4 with the fixed effects of diet and PND, the 2-way interactive effect of diet and PND, and the random effect of cohort. If the interactive effect of diet and PND was not significant, data were analyzed using a 1-way ANOVA to assess differences between dietary treatment groups within individual PND. Activity data were analyzed using the MIXED procedure SAS 9.4 with the fixed effects of diet, week, and cycle, and interactive effects of diet by week, week by cycle, and diet by cycle. All data were analyzed for outliers (i.e., defined as having a Studentized residual with an absolute value ≥ 3), and outliers were removed prior to final statistical analysis. Statistical significance was accepted at *P* ≤ 0.05, and data are presented as least-squares means (LSM) with pooled standard errors of the mean (SEM). Significance values for histological outcomes were obtained from Kruskal-Wallis test using the NPAR1WAY procedure of SAS 9.4.

## Results

### Growth and Tolerance

No differences in general pig well-being were noted during daily checks. Diet had no effect (*P* > 0.05) on ADMI, ADG, G:F, or organ growth (Table 2). Diet also had no effect on stool consistency (data not shown).

**Table 2:** Growth performance of pigs receiving experimental milk replacers differing in ARA and DHA fatty acid concentrations^a^r.

	**Dietary treatment**					
**Outcome**	**CON**	**ARA**	**DHA**	**ARA+DHA**	**Pooled SEM**	**Model *P*-value**
**Growth Performance**						
BW, kg						
Initial	1.91	1.90	1.83	1.94	0.086	0.745
Final	4.56	4.72	4.67	5.64	0.705	0.411
PND 3 to PND 29						
ADG, g/d	102	116	104	136	25.2	0.469
ADMI, g/d	839	898	899	977	115.2	0.758
G:F, g BWG:g liquid milk intake	114.2	122.8	109.2	135.0	13.65	0.202
**Organ weights, g/kg BW**						
Duodenum	5.55	4.89	5.11	4.59	0.534	0.305
Jejunum	36.1	37.6	39.8	37.1	3.15	0.543
Ileum	9.09	9.70	9.54	9.15	0.984	0.714
Whole brain	10.8	10.0	10.1	9.1	1.13	0.573
Liver	37.4	38.1	39.0	37.4	2.54	0.825
Spleen	1.94	2.01	1.87	2.00	0.116	0.823
Thymus	1.64	1.75	1.57	1.72	0.143	0.800
SI total length^b^, cm/kg BW	183	178	192	160	12.1	0.286
Duodenum	18.3	17.8	19.2	16.0	1.21	0.287
Jejunum	137	133	144	120	9.1	0.286
Ileum	27.5	26.7	28.8	24.0	1.82	0.286
Crown to rump length, cm/kg BW	9.02	8.50	8.88	7.68	0.727	0.490

a *Values represent least square means of 10–12 pigs per diet. Measured on PND 30. ARA, arachidonic acid; DHA, docosahexaenoic acid; PND, postnatal day; SEM, standard error of the mean; BW, body weight; BWG, body weight gain; ADG, average daily body weight gain; ADMI, average daily milk intake; G:F, feed efficiency; SI, small intestine*.

b *First 10% of total small intestine length was considered duodenum and the last 15% was considered the ileum*.

### Tissue Fatty Acid Analysis

**Figure 1:**
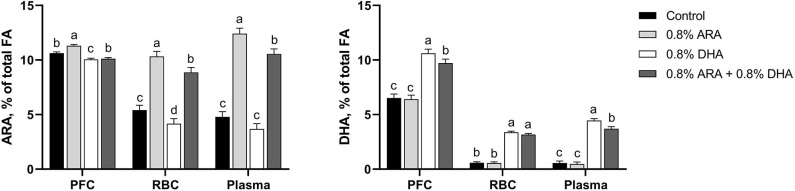
Tissue ARA and DHA concentrations (% of total FA) of pigs receiving experimental milk replacers differing in ARA and DHA fatty acid concentrations. ^a−d^Superscript letters denote treatment means differ (*P* < 0.05). Values represent least square means of 11–12 pigs per treatment. ARA, arachidonic acid; DHA, docosahexaenoic acid; FA, fatty acid; PFC, prefrontal cortex; RBC, red blood cell.

Tissues concentrations of ARA and DHA are shown in Figure 1 and detailed FA profiles are available in Supplementary Tables 1–3. Overall differences (*P* < 0.001) in both ARA and DHA concentrations (% of total FA by weight) were observed in the PFC, RBC, and plasma samples.

#### PFC

In the PFC, pigs fed the ARA diet had higher (*P* < 0.001) percentages of ARA than those fed any other diet. Pigs fed CON and ARA+DHA had had similar percentages of ARA (*P* = 0.140), both of which were higher than those fed DHA (*P* < 0.001 and *P* = 0.015, respectively). Pigs fed DHA alone had higher percentages of DHA than those on any other diet (*P* ≤ 0.006). Those fed ARA+DHA also had a higher percentage of DHA than those fed CON and ARA (*P* < 0.001), while those fed CON and ARA had comparable levels (*P* = 0.742).

Linoleic acid (18:2n-6) and dihomo-γ-linolenic (20:3n-6) percentages were lower (*P* < 0.01) in pigs fed ARA and ARA+DHA than those fed CON and DHA. Adrenic acid (22:4n-6) percentages were different between all treatment groups (*P* < 0.01); pigs fed ARA had the highest percentages, followed by CON, ARA+DHA, and DHA. Percentages of n-6 docosapentaenoic acid (22:5n-6) were higher in pigs fed CON and ARA than those fed DHA and ARA+DHA (*P* < 0.001). Eicosapentaenoic acid (EPA; 20:5n-3) was only present at very low levels, and no differences were observed between treatment groups. Percentage of n-3 docosapentaenoic (22:5n-3) was highest (*P* < 0.001) in pigs fed CON, followed by pigs fed ARA, who had higher (*P* < 0.001) levels than those fed DHA and ARA+DHA; pigs fed DHA and ARA+DHA had comparable percentages.

#### RBC

In RBC, pigs fed the ARA diet had a higher percentage of ARA than those fed any other diet (*P* ≤ 0.012). Pigs fed the ARA+DHA diet also had a higher percentage of ARA than those fed CON and DHA (*P* < 0.001), and those fed CON had a higher percentage than those fed DHA (*P* = 0.029). Pigs fed DHA and ARA+DHA did not differ in their RBC DHA levels (*P* = 0.163), as well as those fed CON and ARA (*P* = 0.865), but those on the two former had higher percentages than the two latter (*P* < 0.001).

Percentage of 18:2n-6 was lower (*P* < 0.001) in pigs fed ARA and ARA+DHA than those fed CON and DHA. Percentage of 18:3n-6 was lower (*P* < 0.03) in pigs fed DHA alone than those fed CON and ARA; pigs fed ARA+DHA had comparable percentages to all other treatment groups. Pigs fed ARA had the highest (*P* < 0.01) percentage of 22:4n-6, pigs fed DHA had the lowest (*P* < 0.01), and pigs fed CON and ARA+DHA had comparable intermediary percentages. Percentage of 22:5n-6 was highest in those fed ARA (*P* < 0.02), followed by those fed CON who had a higher percentage of 22:5n-6 than those fed either DHA or ARA+DHA (*P* < 0.04); pigs fed DHA and ARA+DHA had similar percentages of 22:5n-6. Pigs fed DHA had a higher (*P* < 0.02) percentage of 20:5n-3 than those fed any other diets and those fed ARA had lower (*P* = 0.04) percentage 20:5n-3 than those fed CON. Pigs fed CON and ARA had a higher (*P* < 0.05) percentage of 22:5n-3 than those fed DHA and ARA+DHA.

#### Plasma

In plasma, pigs on the ARA diet had a higher (*P* ≤ 0.009) percentage of ARA than those in any other group, followed by pigs on the ARA+DHA diet, whose percentages were higher than those on both CON and DHA diets (*P* < 0.001). Pigs fed CON and DHA diets had comparable ARA levels (*P* = 0.109). Pigs on the DHA diet had higher percentages of plasma DHA than those on any other diet (*P* ≤ 0.007), followed by the pigs on the ARA+DHA diet, whose levels were higher than those on CON and ARA diets (*P* < 0.001). Pigs on CON and ARA diets had similar percentages of plasma DHA (*P* = 0.751).

Percentage of 18:2n-6 was lower in pigs receiving ARA and ARA+DHA than those fed CON and ARA+DHA (*P* < 0.001). Pigs fed ARA had the highest (*P* < 0.01) percentage of 22:4n-6, pigs fed DHA had the lowest (*P* < 0.01), and CON and ARA+DHA had similar intermediary 22:4n-6 percentage. Percentage of 22:5n-6 were different between all treatment groups (*P* < 0.05); pigs fed ARA had the highest percentages, followed by CON, ARA+DHA, and DHA. Pigs fed ARA alone had 18:3n-3 percentages higher (*P* = 0.007) than those fed DHA alone. Pigs fed DHA had the highest (*P* < 0.03) percentages of 20:5n-3, those fed ARA had lowest (*P* < 0.05), and those fed CON and ARA+DHA comparable intermediary percentages.

### Activity Data

Activity outcomes are presented in Table 3, with results displayed by week and cycle (i.e., day or night). Pigs fed the ARA and ARA+DHA diets exhibited higher activity, measured as average activity counts per min, week-to-week than those on the CON and DHA diets (*P* < 0.05), with the exception of week 4 where only pigs on the ARA diet had higher activity than those on CON and DHA diets. Differences in activity between groups were only significant (*P* < 0.05) during the day cycle. While there were differences in percent time asleep during weeks 2–4, no consistent trends were apparent week-to-week. In the day cycle, where significant differences in activity were observed, pigs fed the ARA diet exhibited the lowest percent time asleep, while those on the DHA diet had the highest.

**Table 3:** General activity of pigs receiving experimental milk replacers differing in ARA and DHA fatty acid concentrations^e^.

	**Dietary treatment**					***P*****-value**			
					**Pooled**			**Overall**	**Sliced**
**Outcome**	**CON**	**ARA**	**DHA**	**ARA+DHA**	**SEM**	**Diet**	**Week**	**effects**	**effects**
**AC/min**									
Week 1	131.7^c^	189.3^a^	139.6^c^	163.2^b^	8.88	<0.001	<0.001	0.184	<0.001
Week 2	132.7^c^	222.7^a^	160.3^b^	194.6^a^	10.55				<0.001
Week 3	154.3^c^	246.0^a^	179.2^b^	224.2^a^	9.65				<0.001
Week 4	215.6^b^	289.3^a^	208.8^b^	233.6^b^	10.69				<0.001
Day	229.2^c^	369.9^a^	240.6^c^	302.2^b^	7.03	<0.001	<0.001	<0.001	<0.001
Night	88.0	103.8	103.3	105.6	6.64				0.197
**% Sleep**									
Week 1	46.8	46.1	46.0	45.9	1.37	0.012	<0.001	0.002	0.928
Week 2	45.0^c^	45.9^cb^	51.5^a^	49.7^ba^	1.61				0.018
Week 3	54.1^a^	47.9^a^	50.6^a^	43.2^b^	1.48				0.004
Week 4	36.0^c^	42.7^a^	41.8^ab^	37.7^bc^	1.64				0.016
Day	32.3^ab^	27.7^c^	34.9^a^	30.8^bc^	1.06	0.012	<0.001	<0.001	<0.001
Night	57.4^c^	63.7^a^	60.0^b^	57.5^bc^	1.02				0.001

a, b, c, d *Within a week (row), means lacking a common superscript letter differ (P < 0.05)*.

e *Values represent least square means of 3–6 pigs per treatment per week. ARA, arachidonic acid; DHA, docosahexaenoic acid; SEM, standard error of the mean; AC, activity count*.

### Hematological Outcomes

Results from CBC and serum chemistry panels are displayed in Table 4. There were no differences between the dietary treatment groups for any parameter on the CBC panel (*P* > 0.05), and all values fell within the corresponding reference intervals for the pig. From the serum chemistry, a difference in creatine phosphokinase (CPK) level was observed (*P* = 0.028), where pigs fed ARA+DHA exhibited somewhat elevated CPK compared with those on the other three diets (*P* ≤ 0.02), although these values were well within the estimated reference range for pigs of similar age. All hematological outcomes were within or just outside of available reference intervals (62, 63).

**Table 4:** Hematological outcomes pigs receiving experimental milk replacers differing in ARA and DHA fatty acid concentrations^c^.

	**Dietary Treatment**						
**Outcome**	**CON**	**ARA**	**DHA**	**ARA+DHA**	**Pooled SEM**	**Model *P*-value**	**Reference interval^**b**^**
**Total and differential cell counts**							
RBC count, × 10^6^ cells/μL	5.24	5.46	5.21	5.41	0.214	0.711	4.08–8.17
Hemoglobin, g/dL	8.98	9.46	8.87	9.36	0.386	0.517	4.32–13.3
Packed cell volume, %	31.3	32.9	31.5	32.6	1.495	0.691	16–41
MCV, fl	61.6	62.2	61.0	62.0	1.097	0.879	34.2–61.3
MCH, pg	17.1	17.4	17.0	17.4	0.305	0.696	9.4–19.8
MCHC, g/dL	27.8	28.3	28.1	28.8	0.645	0.666	26.5–33.6
Platelets, × 10^3^ platelets/μL	658	578	656	625	40.638	0.471	192–832
WBC count, × 10^3^ cells/μL	13.3	11.3	12.2	10.7	1.629	0.576	5.6–18.5
Segmented neutrophils, %	41.8	39.5	38.5	38.4	5.325	0.903	10.8–70.6
Band neutrophils, %	0.18	0.00	0.100	0.167	0.110	0.554	–
Lymphocytes, %	53.2	54.8	56.6	57.2	5.257	0.869	26.2–82.9
Monocytes, %	3.67	4.91	4.00	4.00	0.747	0.668	1.4–8.3
Eosinophils, %	0.67	0.73	0.54	0.25	0.248	0.502	0–1.9
Basophils, %	0.17	0.00	0.18	0.00	0.107	0.439	0–0.90
**Serum chemistry**							
Creatine, mg/mL	0.600	0.555	0.573	0.625	0.040	0.570	0.51–1.39
BUN, mg/dL	10.6	10.4	12.0	11.5	1.34	0.611	4.0–39
Total protein, g/dL	3.52	3.45	3.61	3.57	0.150	0.875	2.5–6.6
Albumin, g/dL	1.72	1.72	1.81	1.82	0.122	0.898	1.9–4.0
Globulin, g/dL	1.80	1.73	1.83	1.64	0.106	0.543	0.3–1.7^e^
Albumin:globulin ratio	0.992	1.055	1.078	1.220	0.116	0.489	0.7–2.2
Calcium, mg/dL	9.96	10.12	10.08	10.19	0.192	0.260	9.9–12.5^e^
Phosphorus, mg/dL	10.9	12.0	11.3	11.9	0.389	0.131	6.3–11.5^e^
Sodium, mmol/L	146	146	146	148	1.036	0.101	125–147
Potassium, mmol/L	6.82	6.55	6.76	6.94	0.205	0.752	2.9–4.6
Sodium:potassium ratio	21.4	22.5	21.7	21.7	0.582	0.648	–
Chloride, mmol/L	110	109	109	110	0.821	0.781	93–108^e^
Glucose, mg/dL	104	109	108	112	7.325	0.833	34–159
ALP, U/L	497	545	499	498	76.93	0.879	110–1,292
AST, U/L	31.8	34.9	28.3	34.8	3.302	0.431	13–65
GGT, U/L	32.8	34.6	33.8	35.2	2.771	0.925	33–94^e^
Total bilirubin, mg/dL	0.209	0.200	0.146	0.225	0.039	0.508	0–0.2^e^
CPK, U/L	476^a^	410^a^	410^a^	643^b^	52.0	0.020	153–5,427^e^
Cholesterol total, mg/dL	108	108	103	109	10.5	0.866	–
GLDH, U/L	0.600	0.727	0.564	0.536	0.119	0.682	–
Magnesium, mg/dL	3.22	3.18	3.21	3.20	0.131	0.997	–
Triglycerides, mg/dL	55.5	69.9	52.6	60.2	7.461	0.337	–
Anion gap	16.5	16.27	15.82	17.50	1.257	0.798	14–29^e^

a, b *Means lacking a common superscript letter differ (P < 0.05)*.

c *Values represent least square means of 9–12 pigs per diet. Measured on PND 30. ARA, arachidonic acid; DHA, docosahexaenoic acid; PND, postnatal day; SEM, standard error of the mean; RBC, red blood cell; MCV, mean cell volume; MCH, mean cell hemoglobin; MCHC, mean corpuscular hemoglobin concentration; WBC, white blood cell; BUN, blood urea nitrogen; ALP, total alkaline phosphatase; AST, aspartate aminotransferase; GGT, gamma-glutamyl transferase; CPK, creatine phosphokinase; GLDH, glutamate dehydrogenase*.

d *Estimated reference intervals for hematological outcomes for 30 day old pigs, retrieved from Ventrella et al. (62), applies to all values unless otherwise indicated*.

e *Estimated reference intervals for hematological outcomes for 42 day old pigs, retrieved from Cooper et al. (63)*.

### Immune Analyses

**Figure 2:**
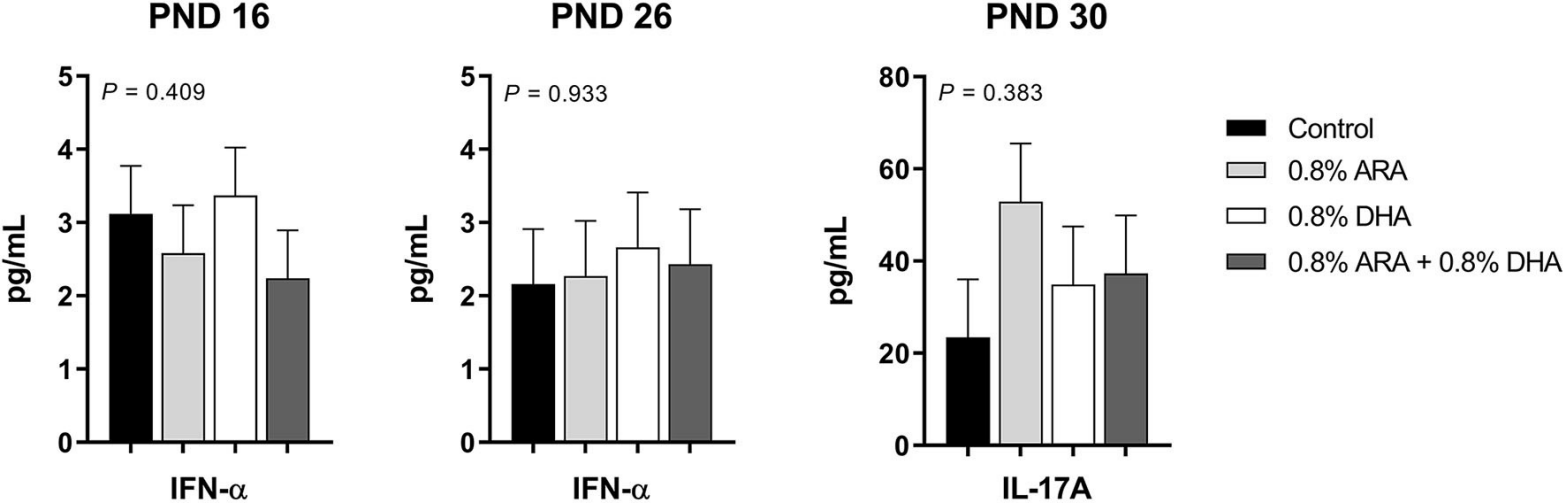
Circulating cytokine levels (pg/mL) of pigs receiving experimental milk replacers differing in ARA and DHA fatty acid concentrations. To stimulant an immune response, all pigs received a two-dose series of KLH via intraperitoneal injection. The first dose (1 mL at 10 mg of KLH/mL) was administered on PND 9 and the second (1 mL at 2 mg of KLH/mL) on PND 19. Plasma from PND 16 and 26 was analyzed for circulating IL-1β, IL-10, IFN-α, IFN-γ, TNF-α, IL-4, and IL-8. Plasma from PND 30 was used to quantify circulating IL-17A. Only those with detectable values are shown. Values represent least square means of 2–4 pigs per treatment for PND 16 and 26, and 7–9 pigs per diet for PND 30. KLH, keyhole limpet hemocyanin; PND, postnatal day; IL, interleukin; ARA, arachidonic acid; DHA, docosahexaenoic acid.

Outcomes from immune parameters are displayed in Table 5. There were no differences (*P* > 0.05) in anti-KLH IgG antibody production between the dietary treatment groups. Additionally, immunophenotyping results suggested that dietary treatment had no effect on the total lymphocyte population size or T cell subpopulations of interest at either time-point (PND 16 or 23). There were also no differences observed in coagulation parameters, TXB_2_, or PGE_2_. No differences were observed in rectal temperatures (data not shown). Results from cytokine analyses are displayed in Figure 2. Nearly all cytokine values fell below the detectable limit. Only those at quantifiable concentrations are displayed, and no differences were observed between dietary treatment groups.

**Table 5:** Immune parameter outcomes of pigs receiving experimental milk replacers differing in ARA and DHA fatty acid concentrations^a^.

	**Dietary treatment**					
**Outcome**	**CON**	**ARA**	**DHA**	**ARA+DHA**	**Pooled SEM**	**Model *P*-value**
**PND 16**						
Total lymphocytes, %	56.6	55.3	46.4	53.0	3.84	0.179
CD3^b^, %	58.5	61.5	53.5	59.6	7.65	0.388
CD3/CD4^c^, %	24.9	27.3	25.8	23.2	3.64	0.466
CD3/CD8^c^, %	15.6	14.2	15.4	15.4	6.10	0.955
CD3/CD4/CD8^c^, %	1.02	1.32	1.30	1.26	0.332	0.725
KLH IgG Ab, ng/mL	2.40	2.38	2.68	2.31	0.191	0.316
**PND 26**						
Total lymphocytes, %	62.2	60.6	51.6	55.0	4.82	0.380
CD3^b^, %	45.8	47.7	41.3	43.9	4.04	0.605
CD3/CD4^c^, %	20.1	19.9	19.0	17.1	2.00	0.698
CD3/CD8^c^, %	20.8	22.6	17.7	25.4	2.66	0.219
CD3/CD4/CD8^c^, %	0.73	1.17	1.07	1.13	0.304	0.247
KLH IgG Ab, ng/mL	2.42	2.80	2.54	2.83	0.209	0.385
**PND 30**						
Fibrinogen, mg/mL	123	116	124	125	8.028	0.760
PT, sec	14.2	14.8	14.4	14.3	0.250	0.082
PTT, sec	13.7	13.9	13.0	13.3	0.494	0.055
TBX2, pg/mL	373.6	340.3	389.5	360.3	36.85	0.280
PGE2, pg/mL	1410.9	1518.8	1420.4	1256.0	172.45	0.733

a *Values represent least square means of 9-12 pigs per diet. To stimulant an immune response, all pigs received a two-dose series of KLH via intraperitoneal injection. The first dose (1 mL at 10 mg of KLH/mL) was administered on PND 9 and the second (1 mL at 2 mg of KLH/mL) on PND 19. ARA, arachidonic acid; DHA, docosahexaenoic acid; PND, postnatal day; SEM, standard error of the mean; KLH, keyhole limpet hemocyanin; Ab, antibody; PT, prothrombin time; PTT, partial thromboplastin time; TXB2, thromboxane-B2; PGE2, prostaglandin E2*.

b *Percent of total lymphocytes that are positive for cell-surface marker CD3*.

c *Percent of CD3-positive lymphocytes that are also positive for cell-surface markers CD4, CD8, or CD4/CD8*.

### Small Intestine Histology

No differences (*P* > 0.05) in intestinal structure were observed except for a slight reduction (*P* = 0.040) in the size score for Peyer's patches in pigs fed DHA alone compared with other dietary treatment groups. Detailed results from histological analyses are provided in Supplementary Table 4.

### Myelination

Results from corpus callosum TEM analysis are displayed in Table 6. No differences (*P* > 0.05) were observed in total, axon, or myelin thickness.

**Table 6:** Corpus callosum genu myelination in pigs receiving experimental milk replacers differing in ARA and DHA fatty acid concentrations^a^.

	**Dietary treatment**					
**Outcome**	**CON**	**ARA**	**DHA**	**ARA+DHA**	**Pooled SEM**	**Model *P*-value**
Total diameter, μm	1.315	1.136	1.099	1.085	0.112	0.482
Axon diameter, μm	0.943	0.815	0.790	0.754	0.094	0.540
Myelin thickness, μm	0.186	0.160	0.155	0.165	0.013	0.391
G-ratio	0.716	0.712	0.719	0.695	0.016	0.723

a *Measures made by transmission electron microscopy. Values represent least square means of 3 pigs per treatment, 300+ axons per sample. Measured on PND 30. ARA, arachidonic acid; DHA, docosahexaenoic acid; PND, postnatal day; SEM, standard error of the mean*.

## Discussion

### Overview

The effect of dietary DHA in the absence of ARA was of primary interest due to the new IF regulations in the European Union. According to these recommendations, IF must contain DHA at levels higher than worldwide breast milk averages, without a requirement for ARA inclusion. This decision has received some resistance, as experts generally agree that ARA should be provided in at least equal or greater concentrations than that of supplemental DHA to mimic the composition of breastmilk (4, 12, 13). Due to the influence of supplemental ARA and DHA on tissue FA incorporation, neural development, and their generally opposing physiological effects on the immune response, we sought to investigate the impact this type of formula could have on developmental outcomes. Diets with no background ARA or DHA were formulated to allow for clear identification of independent and dual impact of ARA and DHA supplementation. Ultimately, our results support several of the concerns raised by the recently published expert opinion regarding IF supplemented with DHA in the absence of supplemental ARA (12). The concerns raised by this group included possible decreases in brain ARA concentrations, as well as potential negative impacts on neurodevelopment, growth, and immunity. Here we validate concerns regarding reduced brain ARA accretion, specifically in the PFC, and highlight potential neurodevelopmental differences that may raise concern and warrant further investigation.

### Fatty Acid Accretion

Both clinical and animal work has demonstrated that plasma, RBC, and cerebral cortex FA concentrations are sensitive to dietary ARA and DHA levels (3, 14, 64, 65). Our results generally align with previous work on ARA and DHA tissue accretion. However, while cerebral cortex ARA concentrations are reportedly more resistant to diet-induced fluctuations, here we observed a decrease in endogenous ARA concentrations in the PFC when DHA was provided in the absence of dietary ARA—thus providing direct evidence to support the view that dietary ARA is required when DHA is included to support tissue accretion in this region. The provision of DHA alone also decreased endogenous ARA concentrations in RBC, whereas ARA alone did not impact endogenous levels of DHA in PFC, plasma, or RBC. Previous work in young pigs has similarly shown that when dietary DHA was provided at 1.0% of total FA, differing dietary ARA concentrations had little to no impact on DHA levels in tissues including the heart, liver, brain, and retina (66). We observed that the combined supplementation lessened the accretion of either ARA or DHA when compared with individual FA supplementation, the only exception being RBC DHA. Similar to infant RBC data report by Colombo et al. ARA levels were reduced when the level of dietary DHA exceeded that of ARA (14). While combined ARA+DHA supplementation reduced DHA accretion in the PFC compared with DHA alone, the inclusion of ARA may be necessary to ensure endogenous ARA concentrations are maintained in this region.

Expectedly, ARA and DHA supplementation also influenced tissue concentrations of several other n-6 and n-3 series PUFA. In young pigs, increasing the dietary ARA and DHA has been shown to reduce 18:2n-6 incorporation in the brain, RBC, and plasma (67). Similarly, here we observed reduced 18:2n-6 concentrations in all three tissue types in pigs receiving ARA or ARA+DHA treatments, suggesting the reduction was primarily driven by dietary ARA content. The inclusion of ARA in the present study also drove down PFC percentages of 20:3n-6, the immediate precursor to ARA in the n-6 desaturation and elongation scheme. Subsequent elongation of ARA yields adrenic acid (22:4n-6), which serves as the third most abundant PUFA inthe brain (68). In each tissue type, the highest percentages of 22:4n-6 were observed in pigs fed ARA alone and lowest in those fed DHA alone. The inclusion of both ARA and DHA attenuated the increase observed with ARA alone and resulted in 22:4n-6 percentage like that of pigs fed CON in both plasma and RBC. Outcomes in 22:5n-6 percentages align with findings from previous piglet work that showed increasing ARA and DHA prompt decreases in 22:5n-6 incorporation in tissues including brain, retina, liver, and RBC (67). Their study reported plasma 22:5n-6 was not as strongly impacted by increasing total ARA and DHA, however, each diet contained similar ARA:DHA ratio. Here, the provision of both ARA and DHA did cause slightly lower 22:5n-6 percentages in plasma, but the largest difference in 22:5n-6 concentrations was observed in pigs fed ARA alone, where the percentage of 22:5n-6 was nearly twice that of the control. Deficiency in n-3 FA and depletion of tissue DHA has been shown to bring about reciprocal replacement with 22:5n-6 (67, 69, 70). Alpha-linolenic acid (18:3n-3) was not detectable in the PFC, nor were differences observed between treatment groups in RBC, but pigs fed ARA alone did exhibit higher plasma 18:3n-3 than those fed DHA alone. EPA (20:5n-3), a precursor to DHA, was only present at very low levels in the PFC of all pigs. Differences in EPA between treatment groups were only observed in the plasma and RBC, where inclusion of DHA alone unsurprisingly drove percentages of EPA up, while ARA alone drove percentages down.

Polymorphisms in the FADS genes influence endogenous ARA and DHA synthesis, and infants with specific genotypes may require different levels of supplementation to maintain adequate status (10, 71, 72). Consequently, the need for dietary ARA inclusion may be more important for infants with certain polymorphisms. Variations in expression of FADS-related genes and the subsequent impact on elongation and desaturation of LA and tissue FA content has also been implicated in the pig (73, 74). While we did not explore specific genetic variations here, this may have contributed to why we observed reductions in ARA concentrations in the PFC, which was in contrast to previous studies that found ARA in the brain was largely impervious to dietary influences. It is important to highlight, however, that observed reductions in ARA concentrations are also likely region-specific.

### Motor Activity

ARA and DHA are present in substantial quantities in regions of the brain involved with motor function. In neonatal baboon brains, the highest DHA concentrations are reported in the globus pallidus, superior colliculus, putamen, and precentralis regions, all of which are implicated in motor functions (24). In neonatal baboons provided moderate or high levels of DHA (0.3 vs. 1.0% of total FA), with constant ARA (0.67% of total FA), ARA concentrations in most neural tissues (cerebral cortex, retina, putamen, caudate, and amygdala) were not impacted by dietary treatment (75). However, differences were observed in the superior colliculus and the globus pallidus. The superior colliculus is a structure involved in visual-motor integration, while the globus pallidus is related to voluntary movement regulation. Animals receiving 1.0% DHA had reduced ARA concentrations in the superior colliculus compared with the 0.3% DHA group and control (no ARA/DHA). Those receiving 1.0% DHA also had reduced ARA in the globus pallidus compared with the 0.3% DHA group, but control ARA levels fell intermediary. Concentrations of ARA in these regions may be particularly sensitive to the diet. DHA at 0.3% of total FA reflects worldwide breast milk concentrations, which range from 0.06 to 1.4% (1). In the present study, DHA was provided at a similar, albeit slightly lower, concentration to that of the high DHA diet in the aforementioned study. We speculate that the provision of DHA at 0.8% of total FA without dietary ARA, as done here, could have caused similar reductions in ARA concentrations in these regions. Alterations in FA concentrations in these regions may have, in part, contributed to the differences observed in activity levels.

Pigs receiving ARA-supplemented diets exhibited higher average activity counts per minute than those not receiving dietary ARA. These patterns of increased activity were consistent with those observed in spontaneous movement measures in rodents supplemented with ARA (31, 32). Harauma et al. reported that mice fed n-3-adequate diets supplemented with an ARA oil (240 mg/kg/day) for 13 weeks exhibited increased spontaneous motor activity compared with those not receiving ARA supplementation (31). In another study by this group using artificially reared delta-6-desaturase knock out mice (D6D-KO), mice unable to endogenously synthesize ARA and DHA from their LA and ALA precursors, mice exhibited the highest spontaneous motor activity levels when they were provided milk supplemented with ARA alone, followed by ARA+DHA, then by DHA alone. As such, only the provision of combined ARA and DHA supplementation in D6D-KO mice diets achieved activity levels comparable to that of wild type control mice (32). In the current study, DHA alone did not trigger changes in activity counts, but the addition of DHA did attenuate the increase observed when ARA was provided. Hence, we believe that the differences in gross motor activity were primarily driven by the increase in dietary ARA.

Differences in activity levels may be due to altered dopaminergic function, as animals on n-3-deficient diets are known to exhibit both increased locomotor activity and alterations in dopaminergic and serotonergic systems (76–78). Dysregulation of the dopamine system is thought to contribute to hyperactivity observed in animals on n-3 deficient diets (78). In newborn rodent brains, Innis and de la Presa Owens reported an inverse relationship between dopamine and phosphatidylserine DHA and phosphatidylethanolamine DHA, whereas a positive relationship was identified between phosphatidylcholine ARA and dopamine levels (79). de la Presa Owens et al. also showed ARA and DHA supplementation to 18:2n-6/18:3n-3-deficient diets normalized concentrations of dopamine, 3,4-dihydroxyphenylacetic (DPOAC), homovanillic acid (HVA), serotonin, and 5-hydroxyindoleacetic acid (5-HIAA) in the pig frontal cortex to that of pigs fed an 18:2n-6/18:3n-3-adequate diet; however, no differences were observed between 18:2n-6/18:3n-3-adequate diets without or with ARA and DHA (80). Conversely, in another study by this group using the pig, ARA and DHA inclusion in 18:2n-6/18:3n-3-adequate diets reduced serotonin in the striatum and elevated dopamine and 5-HIAA in the superior and inferior colliculus (81). Thus, ARA- and DHA-induced alterations in monoamine neurotransmitter concentrations are likely region-specific. Reduced DHA, or rather, an increased ARA:DHA ratio, may have altered FA concentrations in motor regions and contributed to altered dopaminergic and serotonergic function, ultimately influencing gross motor activity as observed in the current study. Further research is warranted to elucidate this response.

### Sleep

Infant sleep and wake patterning can be used in clinical studies to assess central nervous system development and function, and outcomes have been linked to prenatal DHA levels (82–84). Infants born to mothers who were provided a cereal-based DHA food intervention exhibited fewer arousals in quiet and active sleep states during the first day of life than those of mothers who received a placebo (82). During the first 2 days of life, maternal plasma phospholipid DHA levels were also associated with less active sleep and a lower ratio of active sleep to quiet sleep; on day two, infants of mothers with high DHA phospholipids exhibited less sleep-wake transitions and more wakefulness (83). In the present study, actigraphy was used as a proxy measure to quantify movement of pigs during the day and night cycles. While no differences in percent time asleep (i.e., periods lacking in movement) were observed between CON and ARA+DHA during either cycle, the provision of ARA or DHA alone prompted differences during both day and night cycles, highlighting opposing actions and the potential importance of balance between these LCPUFA. During the day, pigs fed DHA exhibited higher percent time asleep, and those fed ARA lower percent time asleep, while the inverse was true for the night cycle. The reduced time spent asleep during the day by pigs fed ARA partially helps explain the markedly higher AC observed during the day cycle.

Prostaglandin D_2_ (PGD_2_) and PGE_2_ are both derived from ARA and are involved in sleep cycle regulation, promoting and suppressing sleep, respectively (33). The present study did not observe any differences in circulating PGE_2_ and did not quantify PGD_2_ levels. Additionally, both ARA and DHA are found in the pineal gland, the brain structure responsible for the production of melatonin, a sleep cycle regulating hormone (34). Lavialle et al. found that compared with controls, hamsters receiving n-3 deficient diets exhibited increased ARA:DHA in the pineal gland, hyperlocomotion associated with striatal hyperdopaminergic, and markedly lower nocturnal pineal melatonin (34). These findings align with the earlier discussion regarding n-3 deficient diets, increased locomotor activity, and alterations in the dopaminergic system. Earlier rodent studies have also demonstrated that the pineal gland composition and function are sensitive to dietary DHA intake (35, 36). Zaouali-Ajina et al. reported that the provision of dietary DHA increased pineal DHA concentrations in rats provided either n-3 sufficient or deficient diets; they also showed that providing DHA to n-3 deficient rats increased levels of nighttime urinary 6-sulfatoxymelatonin, a metabolite of melatonin, to levels similar to n-3 sufficient rodents (35). Neither Zaouali-Ajina et al. nor Lavialle et al. observed a difference in daytime/light cycle pineal melatonin or urinary metabolite concentrations. Conversely, Zhang et al. reported elevated daytime pineal melatonin in rodents on n-3 deficient diets (36). In the present study, alternations in melatonin release and circadian rhythms may have contributed to differences in total sleep quantified during day and night cycles. Differences in measured sleep time could be influenced by sleep patterns or quality, but the present study did not evaluate these specifically. Nonetheless, these results continue to support the need for optimal dietary ARA:DHA ratios as they relate to functional early life outcomes such as sleep quality, quantity, and regulation.

### Myelin

Measures of myelin thickness were explored as a potential mechanism by which dietary ARA and DHA may influence functional outcomes. While no differences in myelin thickness were observed, measures were only made within the corpus callosum and at a single time-point due to protocol constraints. If present, alterations in myelination are likely region-specific, similar to what has been observed with ARA and DHA accretion in the brain. Thus, the timing and location of measurements here may not have been appropriate to detect differences in myelin thickness. Neuroimaging procedures can help provide insight into regions where myelin may be sensitive to diet alterations. Diffusor tensor imaging (DTI) provides indirect measures associated with degree of myelination, including fractional anisotropy, radial diffusivity, and axial diffusivity. In adolescent humans, McNamara et al. utilized DTI to demonstrate a positive linear correlation between RBC DHA and axial diffusivity in the corpus callosum (37). Later, in rats, McNamara et al. showed that reducing dietary DHA and n-3 FA reduced forebrain DHA accretion, which corresponded to reduced adult brain white matter integrity in regions including the right and left external capsules and the corpus callosum genu, as measured by DTI (38). Our lab has previously employed DTI techniques to evaluate brain development in the young pig in response to various dietary interventions. For example, perinatal choline deficiency altered fractional anisotropy in the thalamus and right hippocampus, as well as cerebellar radial diffusivity and mean diffusivity (85). Pigs supplemented with high concentrations of alpha lipoic acid exhibited decreased fractional anisotropy and axial diffusivity in the internal capsule, and iron deficiency resulted in decreased fractional anisotropy values in the caudate, cerebellum, and internal capsule when compared with iron sufficient pigs (86, 87). Assessment of alternate brain regions, such as those described, may prove more successful in detecting differences in myelin characteristics in response to altered ARA and DHA intake.

### Immune Outcomes

The impact of diet on immune function is particularly important during the postnatal period, as newborns have immature adaptive immune systems, limited pathogen exposure, and impaired immunological memory, leaving them especially vulnerable to infections (88). The postnatal period is also an important developmental window for establishing oral tolerance, failure of which may contribute to the development of food allergy (4, 89). ARA and DHA have opposing immunomodulatory effects, and the provision of IF with both ARA and DHA generates a more comparable immune response to that of a breast fed infant (4). Dietary ARA and DHA have been shown to influence immunoregulatory eicosanoid and docosanoid production, T cell function, cytokine production, and B cell activation (3, 43, 90–92). Because of potential modulatory action on immune development and function, the inclusion of high levels of supplemental DHA in IF, relative to average levels currently included in IF and present in breast milk, without the addition of ARA has raised concerns. There is a limited number of studies evaluating the impact of high DHA supplementation levels on early immune development when provided without ARA (3, 4). Tyburczy et al. previously reported on the effects of varying concentrations of dietary ARA and DHA on pig growth and immune response to *M. hyopneumoniae* vaccination (16). They found no differences in intake, growth, clinical chemistry, or hematology parameters. Measures of serum total IgA, IgG, IgM, and *M. hyopneumoniae* antibodies were also not affected, nor were the acute phase proteins, high sensitivity C-reactive protein, haptoglobin, or serum amyloid A. In our study, we utilized an alternative immune stimulant and employed a diet supplemented with DHA in the absence of dietary ARA. Similar to Tyburczy et al. (16), we did not observe differences in antibody production, thus suggesting no alteration of the immune response. We also did not observe any differences in levels of ARA-derived eicosanoids, PGE_2_ and TXB_2_, rectal temperatures, or T cell distributions. Additionally, the only cytokines present at a detectable concentration were IFN-α and IL-17A.

The present study focused on the immune response to an injected stimulant, but additional markers of early immune development could also be explored. Researchers have previously utilized *ex vivo* immune cell stimulation techniques to assess cytokine excretion and characterize T helper (Th) cell subsets (e.g., Th1, Th2, Th17, and Treg) as a factor of immune development (93). Newborns have been shown to exhibit T cell responses that favor Th2 polarization (IL-4, IL-5, IL-10, and IL-13) over Th1 (IFN-γ, IL-2, and TNF-α), but exhibit increasing capacity to produce Th1 cytokines during the first year of life (88, 94). The pig model could also be used to evaluate B cell development, which can be explored using immunophenotyping procedures and various surface cell markers (95). Richard et al. reported that suckling rodent pups consuming maternal milk with higher levels of DHA resulted in higher proportions of activated B cells in splenocyte immune cells (96). Miklavcic et al. found that increasing the dietary ARA in IF from 0.00 to 0.64% of total FA, in the presence of 0.32% DHA, resulted in both reduced proportion of B cells and expression of activation markers (43), again indicating the importance of the dietary ARA to DHA ratio in relation to functional development outcomes.

The immune stimulant, KLH, was selected to help prevent interference from maternal antibodies and has been shown to be safe and effective, eliciting a robust humoral and adaptive response (97). The injection series was chosen to mimic a routine vaccination schedule, and a similar dosing series was effective in eliciting an immune response in the Göttingen minipig (98). While the injection series did elicit a response, as evidenced by antibody production above the estimated baseline, it is possible that KLH administration in the present study did not generate a robust enough immune response to allow measurable differences in antibody production or immune phenotypes. Adjuvants have been included in some previous studies utilizing KLH in the pig to elicit stronger responses, but there is an increased risk for adverse effects with adjuvant inclusion (97, 99, 100). It may be advantageous for future studies to explore alternative, possibly more robust, immune stimulants.

### Hematological Outcomes

The only dietary treatment effect observed in hematological outcomes was for serum creatine phosphokinase (CPK), in which pigs fed ARA+DHA exhibited somewhat elevated levels of CPK compared with those fed other diets. While elevated CPK could be used as a marker of tissue damage, it is fairly non-specific and may be related to the muscle (skeletal or cardiac) or kidney damage. Elevated CPK levels are often recorded after physical exercises (101). The levels observed in pigs fed ARA+DHA were well within the estimated reference range for similarly aged pigs (63). Moreover, we did not observe any indications of tissue damage. This, paired with the lack of any other clinical findings, leads us to believe the elevated CPK levels are not clinically relevant.

### Independent Inclusion of ARA and DHA

There is limited literature on the safety and physiological response to an IF supplemented with ARA in the absence of dietary DHA. Using the young pig model, de la Presa Owens et al. observed similar body and organ growth between pigs consuming a formula with 0.8% ARA alone, a formula with 0.3% DHA alone, an unsupplemented formula, and those receiving a sow's milk control containing both ARA and DHA (102). Using an *in vitro* assay, they also found that formula-fed pigs receiving ARA exhibited similar FADS2 activity toward 18:2n-6 or 18:3n-3 compared with unsupplemented pigs or those receiving DHA. Additionally, ARA inclusion resulted in higher FADS1 activity toward both 18:2n-6 and 18:3n-3 compared with unsupplemented pigs. Huang and Craig-Schmidt demonstrated that the provision of ARA and DHA alone in young pigs resulted in higher and lower *ex vivo* lung eicosanoid production, respectively, than that of pigs fed a combined supplementation, which fell intermediary (103). To our knowledge, the present study is one of the first to assess the impact of ARA supplementation in the absence of dietary DHA with safety and immune response as pivotal outcomes. Here, we did not observe any negative impacts on immune parameters, growth, hematological outcomes, serum chemistry, or small intestine histology when ARA was provided at this level to a diet devoid of dietary DHA. Nor did ARA at this level reduce endogenous DHA concentrations in plasma, RBC, or PFC, but it did appear that provision of ARA may have altered activity patterns. Thus, we conclude that while not likely to be used in practice, no immediate safety concerns were identified with the dietary provision of ARA alone at this concentration.

Similarly, few studies have looked at the safety of supplementing DHA in the absence of dietary ARA on immune response and brain accretion. In the present study, we did not observe any negative impacts on the immune parameters, growth, hematological outcomes, serum chemistry, or small intestine histology when DHA was provided at this level in a diet devoid of dietary ARA. However, supplementation with DHA alone reduced endogenous ARA concentrations in RBC and PFC compared with the control levels.

### Limitations

While the use of a soy-based diet was necessary to achieve a formulation devoid of background ARA or DHA, it is a limitation of the present study. One concern regarding a soy-based diet is its relatively high content of 18:2n-6. Dietary essential FA 18:3n-3 and 18:2n-6 compete for the same desaturation and elongation enzymes to produce DHA and ARA, respectively. Consequently, an excessive 18:2n-6 intake, or an increased ratio of 18:2n-6 to 18:3n-3, may reduce the conversion of 18:3n-3 to DHA (21). However, our study utilized soy protein isolate, which did not contribute a substantial amount of fat to the overall diet. Moreover, all diets contained similar 18:2n-6 concentrations, constituting ~5% of total FA. In addition, 18:3n-3 was provided at ~0.5% of total FA, resulting in 18:2n-6 to 18:3n-3 ratios comparable to those reported in previous studies investigating ARA and DHA supplementation in young pigs (66, 67). The use of the young pig model provided the benefit of more comparable essential FA metabolism to that of a human infant than rodents, but the conversion of essential fatty acids to ARA and DHA are less clearly defined in the pig (55, 56, 104). Another concern may be immunogenicity or estrogen-like activity of dietary soy ingredients, but both of these potential confounds were addressed through the use of soy protein isolate that afforded low allergenicity and contained extremely low isoflavone concentrations compared with other soy-based ingredients.

## Conclusion

The aim of this trial was to evaluate the effect of individual or combined ARA and DHA supplementation on developmental outcomes in the young pig, including body and organ growth, gastrointestinal structure, immune function, general activity, myelin thickness, and fatty acid composition of pertinent tissues. We did not observe any differences in growth outcomes, diet tolerance, or immune parameters. Concentrations of ARA and DHA in the PFC, RBC, and plasma were sensitive to dietary intake when compared with diets devoid of these fatty acids. Results demonstrate that endogenous ARA levels in the PFC and RBC are reduced when only DHA supplementation is provided in the absence of dietary ARA. The provision of ARA supplementation when DHA was provided was necessary to maintain endogenous ARA concentrations in the PFC. Differences in activity levels were noteworthy and demonstrate that dietary ARA and DHA have a functional impact on gross motor activity levels. Based on ARA tissue incorporation, these results support the case for ARA inclusion when supplemental DHA is provided.

## Data Availability Statement

The datasets for this article are not publicly available because the research was funded by a commercial partner. Requests to access the datasets should be directed to Ryan N. Dilger, rdilger2@illinois.edu.

## Ethics Statement

The animal study was reviewed and approved by Institutional Animal Care and Use Committee (IACUC), University of Illinois at Urbana-Champaign.

## Author Contributions

RD, ID, NS, and CB were involved in conceptualization and methodology. KH conducted the animal study and wrote the manuscript. KH, RD, SF, and BS were involved in data acquisition, and analysis, and interpretation. VG conducted microscopy analyses. EB conducted fatty acid analyses. All authors contributed to manuscript edits and revisions.

## Conflict of Interest

ID, NS, and EB were employed by the company DSM Nutritional Products. CB and VG were employed by company Bolder BioPATH, Inc. The remaining authors declare that the research was conducted in the absence of any commercial or financial relationships that could be construed as a potential conflict of interest.

## Abbreviations

ALA: Alpha-linolenic acid
ARA: Arachidonic acid
AC: Average activity count
ADG: Average daily body weight gain
ADMI: Average daily milk intake
CON: Control
DHA: Docosahexaenoic acid
FA: Fatty acid
FADS: Fatty acid desaturase
Fib: Fibrinogen
IF: Infant formula
IQ: Intelligence quotient
IL: Interleukin
KLH: Keyhole limpet hemocyanin
LA: linoleic acid
LCPUFA: Long chain polyunsaturated fatty acid
MR: Milk replacer
PBMC: Peripheral blood mononuclear cells
PNCL: Piglet Nutrition and Cognition Laboratory
PTT: Partial Thromboplastin Time
PND: Postnatal day
PFC: Prefrontal cortex
PGE_2_: Prostaglandin E2
PT: Prothrombin Time
PUFA: Polyunsaturated fatty acid
RBC: Red blood cell
T_X_B_2_: Thromboxane-B2
TEM: Transmission electron microscopy.
