# Supplementary material for: Impact of Arachidonic and Docosahexaenoic Acid Supplementation on Neural and Immune Development in the Young Pig
Source: Front Nutr. 2020 Oct 29;7:592364. doi: 10.3389/fnut.2020.592364 (PMC7658628; doi:10.3389/fnut.2020.592364)
Supplement: Supplementary file 1 [file Table_1.DOCX]

| **Supplemental Table 1.** PFC fatty acids of pigs receiving experimental milk replacers differing in ARA and DHA fatty acid concentrations, % of total FA^1^ | | | | | | |
| --- | --- | --- | --- | --- | --- | --- |
|  | **Dietary Treatment** | | | | **Pooled**  **SEM** | **Model**  ***P*-value** |
| **Outcome** | **CON** | **ARA** | **DHA** | **ARA+DHA** |  |  |
| 14:0 | 1.13 | 1.09 | 1.06 | 1.04 | 0.043 | 0.116 |
| 16:0 | 22.53 | 22.49 | 22.13 | 21.91 | 0.343 | 0.140 |
| 16:1 | 1.22 | 1.15 | 1.24 | 1.15 | 0.069 | 0.448 |
| 16:2 | 0.70 | 0.38 | 0.55 | 0.60 | 0.134 | 0.372 |
| 17:0 | 0.23 | 0.26 | 0.24 | 0.24 | 0.039 | 0.908 |
| 18:0 | 20.19 | 20.21 | 20.27 | 20.11 | 0.117 | 0.809 |
| 18:1 n-9 | 12.71 | 12.59 | 12.75 | 13.04 | 0.376 | 0.422 |
| 18:1 n-7 | 4.01 | 3.92 | 3.81 | 3.96 | 0.068 | 0.173 |
| 18:2 | 0.97^a^ | 0.79^b^ | 0.98^a^ | 0.73^b^ | 0.043 | < 0.001 |
| 18:3 n-6 | 0.02 | 0.01 | 0.02 | < 0.01 | 0.006 | 0.144 |
| 18:3 n-3 | ND | ND | ND | ND | - | - |
| 20:0 | 0.17 | 0.16 | 0.18 | 0.18 | 0.011 | 0.113 |
| 20:1 n-9 | 0.19 | 0.21 | 0.18 | 0.19 | 0.049 | 0.968 |
| 20:2 | 0.31 | 0.26 | 0.28 | 0.22 | 0.066 | 0.737 |
| 20:3 n-6 | 0.51^b^ | 0.43^c^ | 0.57^a^ | 0.43^c^ | 0.014 | < 0.001 |
| 20:4 n-6 | 10.63^b^ | 11.30^a^ | 10.05^c^ | 10.42^b^ | 0.113 | < 0.001 |
| 20:5 n-3 | 0.01 | < 0.01 | 0.01 | 0.01 | 0.004 | 0.161 |
| 22:0 | 0.14 | 0.10 | 0.15 | 0.14 | 0.028 | 0.110 |
| 22:1 | 0.03 | 0.02 | 0.02 | 0.03 | 0.010 | 0.857 |
| 22:4 n-6 | 5.32^b^ | 5.79^a^ | 4.43^d^ | 4.82^c^ | 0.169 | < 0.001 |
| 22:5 n-6 | 5.99^a^ | 6.44^a^ | 4.05^b^ | 3.94^b^ | 0.188 | < 0.001 |
| 22:5 n-3 | 0.25^a^ | 0.21^b^ | 0.16^c^ | 0.15^c^ | 0.011 | < 0.001 |
| 22:6 n-3 | 6.52^c^ | 6.41^c^ | 10.62^a^ | 9.72^b^ | 0.370 | < 0.001 |
| 24:0 | ND | ND | ND | ND | - | - |
| 24:1 | 0.14 | 0.12 | 0.18 | 0.17 | 0.037 | 0.196 |
| ^a-d^Means lacking a common superscript letter differ (*P* < 0.05). | | | | | | |
| ^1^Values represent least square means of 11-12 pigs per treatment. Measured on PND 30. Abbreviations: ARA, arachidonic acid; DHA, docosahexaenoic acid; SEM, standard error of the mean. PND, postnatal day; ND = not detectable. | | | | | | |

| **Supplemental Table 2.** RBC fatty acids of pigs receiving experimental milk replacers differing in ARA and DHA fatty acid concentrations, % of total FA^1^ | | | | | | |
| --- | --- | --- | --- | --- | --- | --- |
|  | **Dietary Treatment** | | | | **Pooled SEM** | **Model**  ***P*-value** |
| **Outcome** | **CON** | **ARA** | **DHA** | **ARA+DHA** |  |  |
| 12:0 | 0.67 | 0.41 | 0.44 | 0.60 | 0.128 | 0.269 |
| 14:0 | 4.16^a^ | 3.64^b^ | 3.92^ab^ | 3.69^b^ | 0.178 | 0.014 |
| 14:1 | 0.00^b^ | 0.00^b^ | 0.04^ab^ | 0.05^a^ | 0.016 | 0.037 |
| 15:0 | 0.10 | 0.14 | 0.11 | 0.12 | 0.021 | 0.705 |
| 16:0 | 26.81 | 27.42 | 27.41 | 27.99 | 0.408 | 0.221 |
| 16:1 | 1.76^a^ | 1.38^bc^ | 1.48^b^ | 1.16^c^ | 0.101 | < 0.001 |
| 17:0 | 0.29 | 0.30 | 0.30 | 0.28 | 0.011 | 0.356 |
| 18:0 | 9.38 | 9.74 | 8.87 | 9.28 | 0.298 | 0.156 |
| 18:1 n-9 | 23.23^a^ | 21.28^b^ | 23.09^a^ | 21.87^b^ | 0.534 | < 0.001 |
| 18:1 n-7 | 1.78^a^ | 1.55^b^ | 1.66^ab^ | 1.37^c^ | 0.073 | < 0.001 |
| 18:2 | 19.98^a^ | 16.86^b^ | 19.07^a^ | 16.03^b^ | 0.409 | < 0.001 |
| 18:3 n-6 | 0.24^a^ | 0.23^a^ | 0.13^b^ | 0.17^ab^ | 0.031 | 0.040 |
| 18:3 n-3 | 0.48 | 0.43 | 0.47 | 0.43 | 0.027 | 0.352 |
| 20:0 | 0.07 | 0.10 | 0.07 | 0.08 | 0.028 | 0.803 |
| 20:1 n-9 | 0.02 | 0.03 | 0.02 | 0.02 | 0.021 | 0.846 |
| 20:2 | 0.12 | 0.07 | 0.12 | 0.07 | 0.038 | 0.614 |
| 20:3 n-6 | 0.54 | 0.59 | 0.56 | 0.58 | 0.029 | 0.525 |
| 20:4 n-6 | 5.41^c^ | 10.33^a^ | 4.17^d^ | 8.87^b^ | 0.461 | < 0.001 |
| 20:5 n-3 | 0.23^b^ | 0.13^c^ | 0.34^a^ | 0.21^bc^ | 0.034 | 0.001 |
| 22:0 | 0.32 | 0.35 | 0.28 | 0.33 | 0.035 | 0.526 |
| 22:4 n-6 | 0.73^b^ | 1.06^a^ | 0.43^c^ | 0.66^b^ | 0.051 | < 0.001 |
| 22:5 n-6 | 0.48^b^ | 0.64^a^ | 0.29^c^ | 0.34^c^ | 0.047 | < 0.001 |
| 22:5 n-3 | 0.48^a^ | 0.50^a^ | 0.41^b^ | 0.41^b^ | 0.024 | 0.009 |
| 22:6 n-3 | 0.58^b^ | 0.55^b^ | 3.38^a^ | 3.17^a^ | 0.107 | < 0.001 |
| 24:0 | 0.51 | 0.56 | 0.44 | 0.52 | 0.112 | 0.913 |
| 24:1 | 0.22 | 0.25 | 0.24 | 0.17 | 0.053 | 0.689 |
| ^a-d^Means lacking a common superscript letter differ (*P* < 0.05). | | | | | | |
| ^1^Values represent least square means of 11-12 pigs per treatment. Measured on PND 30. Abbreviations: RBC, red blood cells; ARA, arachidonic acid; DHA, docosahexaenoic acid; SEM, standard error of the mean; ND, not detectable; PND, postnatal day. | | | | | | |

| **Supplemental Table 3.** Plasma fatty acids of pigs receiving experimental milk replacers differing in ARA and DHA fatty acid concentrations, % of total FA^1^ | | | | | | |
| --- | --- | --- | --- | --- | --- | --- |
|  | **Dietary Treatment** | | | | **Pooled SEM** | **Model**  ***P*-value** |
| **Outcome** | **CON** | **ARA** | **DHA** | **ARA+DHA** |  |  |
| 12:0 | 0.23 | 0.13 | 0.21 | 0.26 | 0.083 | 0.706 |
| 14:0 | 4.31 | 4.13 | 4.42 | 3.90 | 0.295 | 0.606 |
| 15:0 | 0.08 | 0.11 | 0.10 | 0.09 | 0.018 | 0.679 |
| 15:1 | 0.22 | 0.19 | 0.32 | 0.20 | 0.085 | 0.682 |
| 16:0 | 22.05 | 22.55 | 22.81 | 22.68 | 0.377 | 0.486 |
| 16:1 | 2.66^a^ | 2.02^b^ | 2.16^ab^ | 1.66^b^ | 0.204 | 0.008 |
| 17:0 | 0.20 | 0.23 | 0.22 | 0.23 | 0.013 | 0.088 |
| 18:0 | 14.01^ab^ | 14.77^a^ | 13.30^b^ | 14.34^a^ | 0.281 | 0.005 |
| 18:1 n-9 | 19.58^a^ | 17.20^b^ | 18.76^a^ | 17.63^b^ | 0.420 | < 0.001 |
| 18:1 n-7 | 1.99^a^ | 1.72^ab^ | 1.74^ab^ | 1.46^b^ | 0.124 | 0.010 |
| 18:2 | 25.37^a^ | 20.03^b^ | 24.75^a^ | 20.40^b^ | 0.963 | < 0.001 |
| 18:3 n-6 | 0.65 | 0.52 | 0.39 | 0.49 | 0.069 | 0.082 |
| 18:3 n-3 | 0.60^ab^ | 0.48^a^ | 0.66^b^ | 0.56^ab^ | 0.045 | 0.049 |
| 20:0 | 0.00 | 0.01 | 0.00 | 0.01 | 0.006 | 0.408 |
| 20:1 n-9 | ND | ND | ND | ND | - | - |
| 20:2 | 0.67^a^ | 0.21^b^ | 0.24^b^ | 0.09^b^ | 0.082 | < 0.001 |
| 20:3 n-6 | 0.50 | 0.54 | 0.56 | 0.54 | 0.068 | 0.921 |
| 20:4 n-6 | 4.80^c^ | 12.42^a^ | 3.68^c^ | 10.55^b^ | 0.495 | < 0.001 |
| 20:5 n-3 | 0.32^b^ | 0.18^c^ | 0.48^a^ | 0.32^b^ | 0.052 | 0.002 |
| 22:0 | < 0.01 | 0.01 | < 0.01 | < 0.01 | 0.005 | 0.168 |
| 22:4 n-6 | 0.31^b^ | 0.71^a^ | 0.16^c^ | 0.37^b^ | 0.030 | < 0.001 |
| 22:5 n-6 | 0.33^b^ | 0.62^a^ | 0.12^d^ | 0.22^c^ | 0.036 | < 0.001 |
| 22:5 n-3 | 0.33 | 0.39 | 0.31 | 0.32 | 0.036 | 0.329 |
| 22:6 n-3 | 0.56^c^ | 0.47^c^ | 4.45^a^ | 3.71^b^ | 0.186 | < 0.001 |
| 24:0 | 0.20^a^ | 0.17^a^ | 0.05^b^ | 0.03^b^ | 0.029 | < 0.001 |
| 24:1 | < 0.01 | 0.05 | 0.01 | < 0.01 | 0.017 | 0.059 |
| ^a-d^Means lacking a common superscript letter differ (*P* < 0.05). | | | | | | |
| ^1^Values represent least square means of 11-12 pigs per treatment. Measured on PND 30. Abbreviations: ARA, arachidonic acid; DHA, docosahexaenoic acid; SEM, standard error of the mean; ND, not detectable; PND, postnatal day. | | | | | | |

| **Supplemental Table 4**. Intestinal structure of pigs receiving experimental milk replacers differing in ARA and DHA fatty acid concentrations^1^ | | | | | | | | | |
| --- | --- | --- | --- | --- | --- | --- | --- | --- | --- |
|  | **Dietary Treatment** | | | | | | | |  |
|  | **CON** | | **ARA** | | **DHA** | | **ARA+DHA** | | **Model** |
| **Outcome** | **Mean** | **SD** | **Mean** | **SD** | **Mean** | **SD** | **Mean** | **SD** | ***P*-value** |
| *Duodenum* |  |  |  |  |  |  |  |  |  |
| Lymphoid infiltration | 2.7 | 0.5 | 2.5 | 0.7 | 2.5 | 0.7 | 2.8 | 0.4 | 0.839 |
| Neutrophilic/eosinophilic infiltration | 0.9 | 0.3 | 0.8 | 0.6 | 0.8 | 0.3 | 0.9 | 0.5 | 0.782 |
| Hemorrhage | 0.4 | 0.6 | 0.2 | 0.6 | 0.2 | 0.3 | 0.1 | 0.2 | 0.148 |
| Villus atrophy | 1.6 | 1.4 | 1.1 | 1.2 | 1.4 | 1.3 | 0.7 | 0.9 | 0.346 |
| Congestion | 3.1 | 0.7 | 2.4 | 0.7 | 2.6 | 0.9 | 3.0 | 0.6 | 0.270 |
| Cumulative pathology | 8.7 | 1.8 | 6.9 | 2.1 | 7.4 | 2.3 | 7.4 | 0.7 | 0.266 |
| Crypt hyperplasia | 2.2 | 1.0 | 1.8 | 1.0 | 2.1 | 0.8 | 1.6 | 0.7 | 0.185 |
| Villus height (µM) | 583 | 225 | 602 | 209 | 563 | 208 | 739 | 158 | 0.267 |
| Crypt depth (µM) | 339 | 90 | 274 | 73 | 291 | 81 | 301 | 50 | 0.626 |
| Villus/crypt ratio | 1.7 | 0.7 | 2.1 | 0.8 | 1.9 | 0.9 | 2.5 | 0.6 | 0.071 |
| Total mucosa thickness (µM) | 923 | 279 | 873 | 258 | 852 | 243 | 1040 | 180 | 0.312 |
| *Jejunum* |  |  |  |  |  |  |  |  |  |
| Lymphoid infiltration | 2.4 | 0.4 | 2.2 | 0.6 | 2.3 | 0.6 | 2.3 | 0.3 | 0.710 |
| Neutrophilic/eosinophilic infiltration | 1.4 | 0.6 | 1.2 | 0.5 | 1.6 | 0.7 | 1.3 | 0.6 | 0.458 |
| Hemorrhage | 0.0 | 0.1 | 0.1 | 0.1 | 0.0 | 0.0 | 0.0 | 0.1 | 0.780 |
| Villus atrophy | 0.4 | 0.7 | 0.5 | 0.9 | 0.4 | 0.7 | 0.1 | 0.3 | 0.589 |
| Congestion | 2.7 | 0.6 | 2.4 | 0.8 | 2.6 | 0.7 | 2.7 | 0.5 | 0.875 |
| Cumulative pathology | 6.9 | 1.2 | 6.2 | 1.9 | 6.7 | 1.9 | 6.4 | 1.0 | 0.347 |
| Crypt hyperplasia | 0.8 | 0.7 | 0.5 | 0.7 | 0.5 | 0.6 | 0.1 | 0.3 | 0.147 |
| Villus height (µM) | 590 | 112 | 631 | 226 | 621 | 208 | 671 | 74 | 0.259 |
| Crypt depth (µM) | 197 | 32 | 180 | 61 | 184 | 54 | 185 | 44 | 0.955 |
| Villus/crypt ratio | 3.1 | 0.8 | 3.3 | 1.0 | 3.4 | 1.4 | 3.8 | 1.0 | 0.482 |
| Total mucosa thickness (µM) | 787 | 114 | 809 | 285 | 805 | 228 | 856 | 96 | 0.309 |
| *Ileum* |  |  |  |  |  |  |  |  |  |
| Lymphoid infiltration | 2.2 | 0.3 | 2.2 | 0.7 | 2.1 | 0.5 | 2.3 | 0.3 | 0.319 |
| Neutrophilic/eosinophilic infiltration | 1.6 | 0.8 | 1.5 | 0.7 | 1.6 | 0.7 | 1.6 | 0.5 | 0.953 |
| Hemorrhage | 0.0 | 0.1 | 0.1 | 0.2 | 0.1 | 0.2 | 0.2 | 0.4 | 0.732 |
| Villus atrophy | 1.0 | 0.8 | 0.7 | 0.9 | 1.2 | 1.2 | 0.6 | 0.8 | 0.471 |
| Congestion | 2.6 | 0.6 | 2.2 | 0.8 | 2.2 | 0.6 | 2.6 | 0.6 | 0.355 |
| Cumulative pathology | 7.5 | 1.6 | 6.6 | 1.7 | 7.1 | 2.1 | 7.3 | 1.3 | 0.806 |
| Crypt hyperplasia | 0.0 | 0.0 | 0.0 | 0.0 | 0.0 | 0.0 | 0.1 | 0.3 | 0.418 |
| PP lymphoid cellularity | 3.9 | 0.4 | 3.7 | 1.0 | 3.3 | 0.9 | 4.1 | 0.3 | 0.040 |
| Villus height (µM) | 555 | 98 | 530 | 183 | 493 | 172 | 605 | 147 | 0.675 |
| Crypt depth (µM) | 151 | 36 | 157 | 49 | 164 | 44 | 167 | 25 | 0.237 |
| Villus/crypt ratio | 3.8 | 1.0 | 3.3 | 1.3 | 3.0 | 1.2 | 3.7 | 1.2 | 0.571 |
| Total mucosa thickness (µM) | 706 | 115 | 685 | 218 | 656 | 192 | 772 | 145 | 0.556 |
| ^1^Values represent numerical means of 10-12 pigs per diet. Villi and crypt morphometrics measured at 100-x in µM (average of 5 representative villi). Measured on PND 30. Lesion Scoring: 0 = normal, 1 = minimal, 2 = mild, 3 = moderate, 4 = marked, 5 = severe. Abbreviations: ARA, arachidonic acid; DHA, docosahexaenoic acid; SD, standard deviation; PP, peyers patch; PND, postnatal day. | | | | | | | | | |
